# The Nature of Client Agency Prior to Therapy: A Qualitative Study on Clients’ Narratives

**DOI:** 10.5334/pb.1104

**Published:** 2022-01-17

**Authors:** Emma Acke, Melissa Miléna De Smet, Kimberly Van Nieuwenhove, Reitske Meganck

**Affiliations:** 1 Department of Psychoanalysis and Clinical Consulting, Ghent University, Henri Dunantlaan 2, 9000 Ghent, BE

**Keywords:** client characteristics, client agency, qualitative research methods

## Abstract

Client agency is considered a crucial contributor to good treatment outcome. Recent studies, however, differ strongly in how they conceptualise and investigate agency. The current study explores the nature of client agency in ten clients’ pre-treatment interviews. Applying Consensual Qualitative Research, we constructed three overarching categories, subdivided into 14 sub-categories capturing both between- and within-person differences in agency before therapy. We found that all participants oscillated between the experience of a lack of grip on problems on the one hand and noticing their involvement in the problem and taking action on the other. These results present a dynamic conceptualisation of client agency. This allows us to ask pertinent questions for both future research and clinical practice.

Decades of efficacy research provided evidence for the effectiveness of psychotherapy in treating various mental health issues such as Major Depressive Disorder (MDD) (Cuijpers, 2015). Generally, psychotherapeutic treatment generates longer lasting effects than medical treatment alone (Cuijpers et al., 2013; Hollon and Ponniah, 2010). Yet, while it is well-established that psychotherapy is effective, to date limited knowledge exists on how psychotherapy works (Cuijpers et al., 2013; Kazdin, 2009). In order to advance the field, further examination of the processes of change is hence needed (Kazdin, 2007). In this regard, especially the role of the client is considered quintessential for understanding how therapy works. As Bohart and Wade (2013, p. 246) state: ‘If clients really do play a central role in therapy outcome, then more research needs to focus on how clients do this’.

The client is widely recognized as the principal contributor to therapeutic progress (Bohart & Tallman, 2010). In his theory of the client as an active self-healer, Bohart (2000) argues that the client’s active engagement with interventions rather than the interventions themselves leads to therapeutic change. Recent studies align with this theory, indicating that clients are active participants in relationally based change processes rather than passive recipients of the treatment implemented by the expert-therapist (Dulsster et al., 2019; Williams & Levitt, 2007). In particular, the client’s agency is considered essential for understanding good therapy outcome and psychological well-being (Adler, Skalina & McAdams, 2008; Adler, 2012; Hobbs & Mclaren, 2009; Irving et al., 2004; Schafer, 1993; Williams & Levitt, 2007). A lack of agency has been put forward as a primary reason for seeking help in psychotherapy (Wahlström, 2006). Particularly in Major Depressive Disorder (MDD), a lack of agency is predominantly present (Hirsch, Sirois & Lyness, 2011). Hence, stimulating a client’s agency is seen as an essential ingredient contributing to change in psychotherapeutic treatment (Adler, 2012; Bohart, 2000; Levitt, Pommerville & Surace, 2016; Williams & Levitt, 2007).

Agency ‘is concerned with affecting things, others, oneself, or one’s life’ (Mackrill, 2009, p. 195). In his article, Mackrill (2009) addresses the conceptual confusion concerning agency in psychotherapy research and offers a model that presents six ways psychotherapy research constructs client agency. In the current study, we approach client agency as constructed in the client’s life course, which aligns with an idiographic, narrative take on agency (Mackrill, 2009). Building on Ochs and Capps (1996, p. 19), we conceptualise narratives as ‘verbalized […] framings of a sequence of actual or possible life events’. Multiple authors acknowledged agency as a core component in such narratives (Adler, 2012; Bamberg, 2011; Hauser et al., 2006). Firstly, Adler (2012) regards agency as an important thematic cluster in persons’ life narratives. In his study, he approaches agency as ‘concerned with the individual’s autonomy, achievement, mastery, and ability to influence the course of his or her life’ (Adler, 2012, p. 368). As such, client agency is akin to the basic human need for control and orientation (Epstein, 1990). In contrast, a lack of agency is understood as having no impact on one’s functioning and life circumstances. When feeling little agentic, a person is positioned as a passive object or victim of their environment (Seilonen & Wahlström, 2016). In his study, Adler (2012) found increases in agency in narratives is related to improvements in mental health. Secondly and likewise, Hauser, Allen and Golden (2006) identified agency as a core feature in narratives of resilient teens. In their book they defined agency as ‘the conviction that what one does matters, that one can intervene effectively in one’s own life’ (Hauser et al., 2006, p. 39). Thirdly, also Bamberg (2011) considers agency, alongside constancy/change and sameness/difference, as a central ‘dilemma’ in identity-constructing narratives. In his theorization, he opposes a binary interpretation of agency that either ascribes control to an ‘agentic I’, or to the outside world that determinates an ‘undergoing me’. Rather, he propounds a continuum on which narrating subjects navigate, with a persons’ agency on the one end and determining structures (e.g. social structures, psychological dispositions) on the other end (Bamberg, 2011).

Thus, agency is considered as a central theme in narratives and is related to improved mental health. Yet, research up until now mostly approaches agency in a quantitative manner (e.g., Adler et al., 2008; Adler, 2012; Hobbs & Mclaren, 2009; Irving, 2004) or with a focus on how nonagency is discursively constructed at the outset of and throughout treatment (e.g., Seilonen and Wahlström, 2016; Toivonen, Kurri and Wahlström, 2020). In spite of the extensive evidence for agency as both a precursor of good therapy outcome as well as a crucial aspect during the therapy process, little research can be found on the nature of agency in clients’ narratives. As such, client agency remains an abstract and difficult to grasp concept. Yet, given its therapeutical importance, it is paramount to investigate how agency appears before (and during) therapy. Thus, a bottom-up account of the nature of (non)agency in narratives prior to treatment could be helpful in further nuancing the conceptualisation of pre-treatment client agency and investigating its appearance in narratives prior to therapy.

Therefore, the current study explores the appearance of client agency in narratives of depressive clients prior to the start of their psychotherapeutic treatment, addressing the following research question: ‘How does client agency appear in narratives prior to psychotherapeutic treatment?’. Since the diagnosis of Major Depressive Disorder (MDD) is associated with a decreased sense of agency (Cheavens, 2000; Huber et al., 2018), this offers us an especially appropriate case to study our research questions. We aim to approach agency from an idiographic vantage point, considering it as a pre-treatment variable that fluctuates between as well as within persons. Via an in-depth, qualitative investigation of clients’ pre-treatment interviews, we constructed a bottom-up, tentative understanding of client agency prior to therapy.

## Method

Given the exploratory nature of the research question, we opted for a qualitative approach departing from the data in a bottom-up manner. Qualitative research focuses on the rich description of phenomena and is characterised by its open and flexible research approach (Timulak and Elliott, 2019). During data analysis, we built on the principles of Consensual Qualitative Research (Hill, 2012). Core characteristics of this method include the triangulation of researchers’ perspectives in approaching the data, the process of discussing analyses until consensus is reached and the use of external auditors to supervise the analyses. Furthermore, this method strives to examine the representativeness of the results across cases (Hill, 2012).

### Participants

The sample of this study consisted of eight women and two men, ranging in age between 29 and 57 (*M* = 42, *SD* = 9.35). All participants were Belgian, White and were diagnosed with Major Depressive Disorder (MDD), drawing on the Structured Clinical Interview for DSM-IV (SCID-I and-II; First, Spitzer, Gibbon and Williams, 2002; First, Gibbon, Spitzer, Williams, and Benjamin, 1997). Other comorbid diagnoses were anxiety disorders (five participants) and personality disorders (four participants). Six participants showed comorbidities with on average two diagnoses apart from MDD. Out of ten participants, eight had previously sought assistance of a psychologist or psychiatrist. These previous therapeutic encounters ranged from consulting several psychologists and psychiatrists over time, going through therapeutic processes of several months to attending only one therapy session. Three participants explained how their previous therapeutic experiences took place a considerable long time ago (more than a decade ago). Three participants were unemployed at the beginning of treatment, two of them due to health circumstances. This study was approved by the Ethical Committee of the University Hospital of Ghent (Belgium; B670201318127). All participants gave informed consent before data was gathered. To protect the participants’ privacy, we refer to the participants using pseudonyms.

### Material

The Clinical Diagnostic Interview (CDI; Westen, 2006) is a semi-structured interview that probes both clinical complaints and a wide range of intra- and interpersonal experiences. This clinical interview prompts the participant to narrate, more specifically about what brings them to therapy and both past and current life experiences and circumstances. These narratives, initiated by the broad, open-ended questions from which the CDI departs, make the interviews particularly suitable to study agency. The duration of the interviews ranged from 41 to 148 minutes and was on average 101 minutes long.

### Procedure

#### Data gathering

Data were gathered within a large-scale RCT on the process and outcome of psychotherapy for MDD (Meganck et al., 2017). Three co-authors took part in the process of data gathering for the initial study and all authors have previously carried out analyses on the gathered data, which optimises the context for secondary analyses (Gladstone, Volpe & Boydell, 2007). Participants for the current study were randomly selected from the total RCT sample (N = 100) until data was saturated (i.e., new cases did not provide new information for data analysis). This aligns with a purposeful-random sampling strategy (Patton, 2002) where one randomly selects participants in a purposely chosen, delineated sample which in the current study consists of the RCT sample. In total, ten participants were randomly selected. Analyses were carried out on the Clinical Diagnostic Interviews (CDI; Westen, 2006), which were conducted prior to therapy. All interviews were transcribed verbatim and pseudonymised before data analysis.

#### Data analysis

Building on the principles of Consensual Qualitative Research (CQR; Hill, 2012), analyses were carried out by the first author in collaboration with the second and third author, with whom each step in the analysis procedure was discussed until consensus was reached. During the process of coding and clustering, the fourth author functioned as an external auditor by asking critical questions, prompting to clarify several categories and make their descriptions denser. Data analysis consisted of three major steps.

In the initial phase of coding (step 1), we remained open to explore whatever the data holds for our research question. In this phase, each coder separately read the interviews thoroughly, selected relevant parts based on the research question and created codes while sticking closely to the participants’ speech. Fragments were selected when narratives reflected the position participants ascribed themselves in experienced complaints or difficulties. Subsequently, the coding of every case was discussed separately until consensus was reached on the relevance to client agency and how agency particularly appeared in that fragment (Hill, 2012). The first phase resulted in interview transcripts with initial codes and associated commentaries.

In the second phase (step 2), the first author tentatively clustered the consented initial coding into meaningful categories for each participant. This resulted in a document per participant including clustered initial codes, memo writing on possible interpretations and relevant quotes extracted from the interview. This document was discussed with the second or third co-author and altered (combining and/or dividing categories, adding new categories and/or new remarks) until consensus was reached (Hill, 2012). The final document consisted of a number (min. 5 – max. 16) of categories that characterised the appearance of agency within that specific participant.

In the last phase (step 3), a categorization concerning agency was built based on the comparison of the clustered coding across the ten participants. The above-described participant-specific categories were clustered into categories transcending all participants based on similarities and differences concerning the appearance of agency. During this last phase, clusters were re-named, split up, combined, etc. in order to heighten the consistency of the different categories and minimise overlap. During the coding and clustering process, it became clear that the three main categories are closely intertwined with each other. Consequently, in a last step, we schematised the categories to visualise their interconnectedness. The prevalence of each category was indicated using the nomenclature of Hill et al. (2005): General: ≥90% of the participants (9–10); Typical: ≥50% and <90% (5–8); Variant: ≥20% and < 50% (2–4); Rare: <20% (1 participant).

#### Quality Control

Several measures were taken to guarantee credibility. The research team conducting the analyses was gathered aiming at a balance in terms of familiarity with the interviews, academic and clinical background and phase in career to ensure a horizontal and open dialogue (Parker, 2007). All authors are in varying degrees familiar with psychodynamic and psychoanalytic theory. This predominant theoretical background inevitably influenced the interpretation of the data and therefore the obtained results. To acknowledge and reflect on the influence of our theoretical background during data analysis, our ideas and interpretations were written down in a reflective diary and preconceptions (e.g., interpretations concerning how agency might be understood and its relations to other variables) were made explicit using memo writing. Moreover, data analysis was triangulated over several interviews and coders. The fourth author functioned as an external audit (i.e., not involved with analysing the interviews); she urged the coders to depart from the collected data, questioned the influence of assumptions in the results, and encouraged the coders to write down psychoanalytic interpretations in a separate diary. Furthermore, we opted for a purposeful random sampling strategy that limits the influence of our preconceptions on case selection. To guarantee reliability, we aimed to describe the process of analysis in a transparent manner (Timulak & Elliott, 2019).

## Results

**Figure 1:**
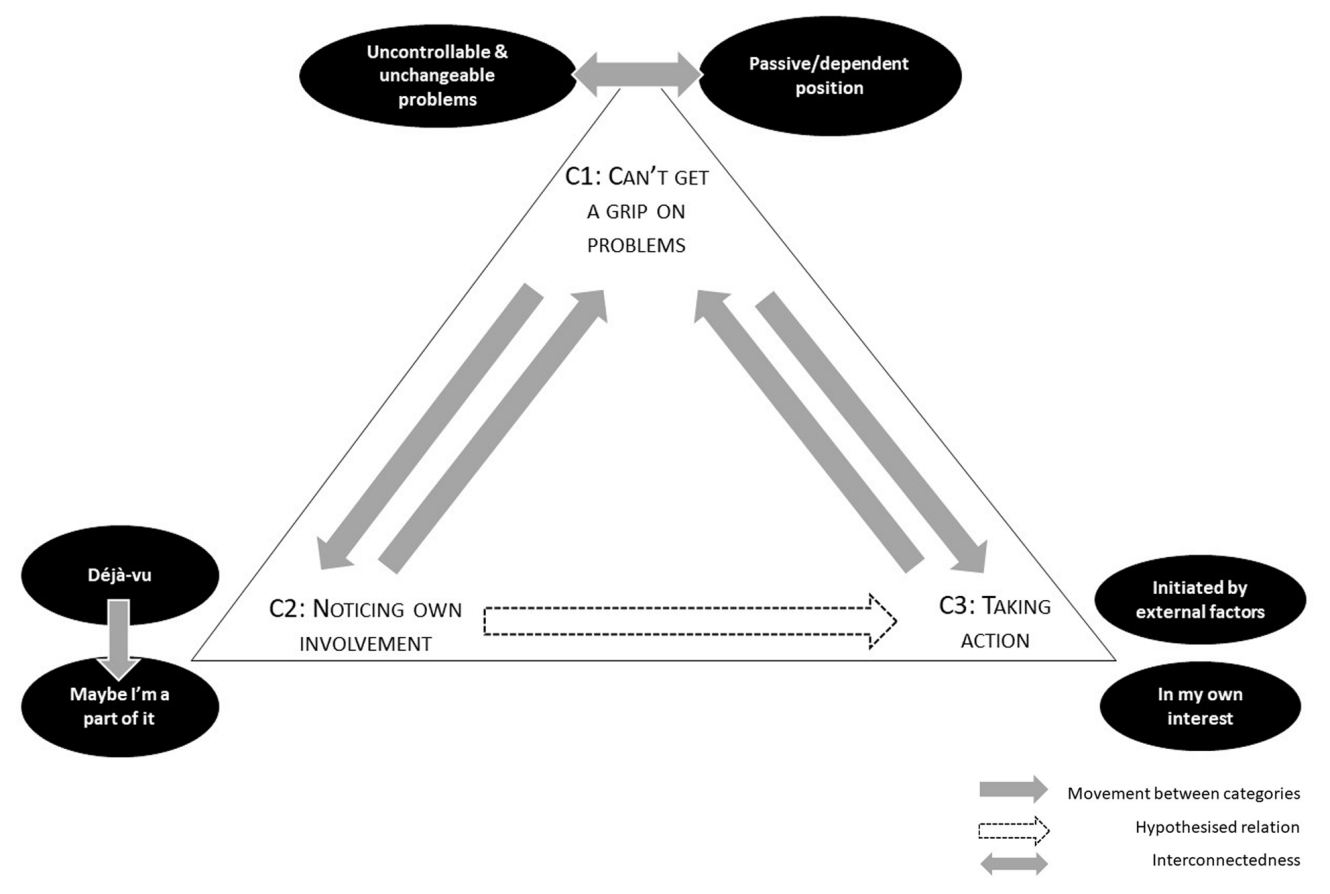
Interrelated categories concerning agency prior to treatment.

This study investigated how agency appears in the participants’ speech prior to therapy. 14 categories resulted from our qualitative analyses and were clustered into three overarching categories. The participants expressed in their narratives that they could not get a grip on their problems (C1), although they noticed their involvement in these problems (C2) and were to some extent able to take action (C3). ***Figure 1*** depicts how all participants oscillated between these different positions, showing a dynamic rather than static nature of clients’ agency prior to treatment. The (sub)categories are summarised in ***Table 1*** and will be described in more detail below.

**Table 1:** Taxonomy of clustered categories concerning agency prior to treatment.


**C1: CAN’T GET A GRIP ON PROBLEMS** **(GENERAL)**						

**Uncontrollable and unchangeable problems** **(General)**				**Passive/dependent position** **(General)**		

General, external circumstances (Variant)	Malevolence of specific others (Typical)	Difficulties within selves (Typical)	Formulated as unchangeable facts (Typical)	Having no impact (Typical)	Dependent position (Typical)	Passive position in therapy (Variant)

**C2: NOTICING OWN INVOLVEMENT** **(GENERAL)**						

**Déjà-vu** **(Typical)**				**Maybe I’m a part of it** **(Typical)**		

**C3: TAKING ACTION** **(TYPICAL)**						

**Initiated by external factors** **(Typical)**				**In my own interest** **(Typical)**		

Worsening of problems (Typical)	Encouragement of others (Rare)			Important life choices (Typical)	Own solutions to handle difficulties (Variant)	Therapy as a tool to get back up (Typical)


*Note*: The prevalence of each category was indicated using the nomenclature of Hill et al. (2005): General: ≥90% of the participants (9–10); Typical: ≥50% and <90% (5–8); Variant: ≥20% and <50% (2–4); Rare: <20% (1 participant).

### Category 1: Can’t Get a Grip On Problems

The first category (C1) comprises two interrelated sub-categories (represented by the double arrow; see ***Figure 1***), subdivided into seven subthemes in total (see ***Table 1***; in the results section all subthemes are written in italics). The participants generally narrated about problems beyond their control. They tended to describe these as unchangeable facts which fostered a rather passive position towards them. On the other hand, this passive position may also perpetuate the experience of problems as being uncontrollable and unchangeable. We consider these subcategories as interrelated, constituting together the feeling of a lack of grip on problems the participants are facing.

#### Uncontrollable and unchangeable problems

Participants generally narrated about various problems they were encountering. These formed the core complaints with which they sought help from a therapist. The participants attributed these problems either to external, uncontrollable factors or to internal, fixed and unchangeable causes.

Regarding the former, participants variantly linked their difficulties to *general, external circumstances* such as the seasons, the weather, ‘the cruel world’, etc. Ariana, for instance, said that the way she is feeling is directly linked to ‘what is going on around her’. Also, participants typically depicted complaints as caused by the *malevolence of specific others* in their environment. They felt like they were surrounded by ‘the wrong people’, who are ‘treating them like trash’. On the other hand, participants typically experienced *difficulties within* them*selves*. They listed situations where they did not recognise themselves (e.g., getting angry easily, being forgetful, etc.), encountered unpredictable physical complaints, and were the object of their impulsivity. Here, the feeling of impotency prevailed, by attributing the problem to a part of themselves they did not (longer) have a grip on: ‘I have to go upstairs when the children make too much noise, otherwise I would begin to roar. I would yell at anyone I encounter. I can’t endure anything; it only gets worse. Nobody knows me like this.’ (Lisa)

Paradoxically, participants typically *formulated* the above-mentioned encountered problems *as unchangeable facts*, assigning them a rather determining role. This was mostly achieved by attributing their problems to static, internal characteristics such as personality, genetics or a diagnosis. Ariana, for instance, attributes her depressive symptoms to a genetical burden: ‘I know it runs in my family, so I’m genetically burdened, and dyslexia comes with it. I’m already glad I don’t have a tendency to be an alcoholic or drug addict.’ One participant, Viviane, did not attribute her problems to an internal characteristic, but rather she described her problems as ‘cultivated’. According to her, a person is ‘the sum of her experiences’.

#### Passive/dependent position

Coinciding with the general belief that problems were both uncontrollable and unchangeable, it seemed that participants displayed a predominant passive position, as such resulting in the general feeling of being unable to get a grip on their problems.

This passive position was expressed by the feeling of *having no impact* on the problems; they came out of nowhere, or the participants did not know what to do about them. George described this feeling as a ‘lack of entrepreneurial spirit’. He states: ‘I’m not an idiot, to put it bluntly, so I know what’s wrong, but, I don’t know what should be done.’ Others were convinced that nothing they could do, would help them anyway. As Ariana stated in the interview: ‘Whatever we do, it’s never any good’.

Besides the feeling of a lack of impact, it was remarkable that participants placed themselves in a *dependent position* towards concrete others in their environment. Typically, participants told us they were dependent on others, who were in most cases romantic partners. Participants mentioned physical dependence due to illness, financial dependence as well as emotional dependence. In Viviane’s view financial dependence doesn’t matter as long as you’re emotionally dependent:

‘I didn’t know I was so dependent, so terribly dependent. I always laugh with economic independence. I am completely economically independent. It has nothing to do with being dependent of your husband […] Why should you be economically independent when you are completely tied to him for everything else? So I didn’t know I was that dependent. I can’t do anything without him.’ (Viviane)

Participants variantly repeated this rather *passive position in* their expectations regarding *therapy*. Here, the therapist was expected to intervene in several ways to alleviate their problems, leaving the participants in a rather impotent position. They, for instance, expect the therapist to give them ‘a label’, to give a ‘kick in the butt’ and to ‘confront them by asking the right questions’. Two participants did not express any, or even negative, expectations, Lisa for instance, stated: ‘I have no idea. I have no expectations. The only thing I know is that I have the feeling that nothing is going to help.’

### Category 2: Noticing Own Involvement

Throughout the interviews, the predominant feeling of lack of grip on problems (C1) was intermittently interrupted by the acknowledgement of their own involvement in the problems participants encountered (C2). The two thick arrows in ***Figure 1*** between ‘can’t get a grip on problem’ and ‘noticing own involvement’ represent how participants moved dynamically between both experiences. The second category is subdivided into two subcategories: on the one hand, recognizing the reiteration of a certain pattern (‘Déjà-vu’) and, on the other hand, questioning one’s contribution to the problems (‘Maybe, I’m a part of it’). Both categories were interwoven in paricipants’ narratives: questions concerning one’s involvement propped up shortly after elaborating on the encountered problems and/or on their repetition. A large variation among participants in the extent to which they elaborated on their involvement was observed.

#### Déjà-vu

The participants typically recognised a certain repetition of encountered problems. One the one hand, they noticed patterns over time. Viviane, for instance, describes how every day she ‘wakes up in the desert’, referring to the experience of fear, emptiness and a lack of perspective, but then calms down as the day progresses: ‘the closer I get to work, the more the fear ebbs away’. On the other hand, they recognised a pattern in the content of their problems. Ariana, for instance, noticed during the interview that she ‘keeps engaging herself with the same kind of characters and keeps attracting them somehow’. Lisa named the re-appearance of the adultery of her romantic partners as a ‘déjà-vu’.

On the one hand, the realisation of a repetition seemed to encourage participants to wonder about their own involvement in the (re)occurrence of certain problems (C2; ‘Maybe I’m a part of it’). On the other hand, participants variantly attributed the possibility for change to others and as such did not feel able to control the reiteration in problems, moving back to a position where a loss of grip on the situation predominates (C1):

‘They say ‘lightning never strikes the same place twice’ but often it strikes me three or four times. So, I would love to see that change in me, that they wouldn’t fool me around anymore. Because [currently] they know they can always count on me. They can hit me in the face today and I’ll kiss them again tomorrow.’ (Claudia).

#### Maybe I’m a part of it

The participants typically mentioned that the problems they are facing may have something to do with themselves. Viviane, for instance, described her involvement in the encountered difficulties as a ‘self-destructive tendency’: ‘It all slipped through my fingers. I gave everything I worked hard for away’. The noticing of an own involvement was variantly preceded by acknowledging a repetition in the encountered problems or by the observation that things were not clearing up, even after their presumed external causes were gone. As Laura says to herself: ‘Come on, it’s all over now. You can re-continue your life. But it doesn’t work like that.’ Moreover, some participants questioned how their past choices and behaviour contributed to the problems. Lisa, for instance, narrates in her interview: ‘It was like a déjà-vu (…) And now I wonder how I could make the same mistake again? How could the same thing happen again?’

Furthermore, participants variantly told us that they actively ‘kept up appearances’ for the outside world, even when things were going bad. They ‘pushed away their emotions’, masked their problems and ‘acted as if nothing happened’. Christina also ‘kept up appearances’ in a previous therapeutic treatment, where she presented herself as helpless and stuck in an unchangeable situation (cf. C1; passive position). She explained:

‘I had a conversation with a psychologist and with a career coach, but I noticed that I was able to explain my situation in such a way that it seemed like I couldn’t do anything about it, which isn’t quite in line with how it was. Maybe I wasn’t open for, uhm, for outside help, and I think I am ready now. Well, I hope I am because I really need it.’ (Christina)

### Category 3: Taking Action

Participants generally narrated how they took action in the past to solve or alleviate their encountered problems. The third category is divided into two subcategories; actions were either initiated by external factors (worsening of situation, encouragement of others), or by the participant’s initiative to ameliorate life circumstances. These two subcategories are divided into five subthemes in total. In their narratives participants moved back and forth between narrating the lack of grip on problems (C1) and the description of taken action in the past (C3). Although we expected actions to be based on a questioning of involvement (C2), we didn’t find a relation between the second and the third category.

#### Initiated by external factors

External factors that initiated actions were, on the one hand, the *worsening of problems* variantly leading to the decision to ‘escape’ in the form of travelling or moving abroad. Although this was helpful for the participants, their decision to move abroad seemed to stem from external necessity rather than their desire: ‘I’m going to be on an aeroplane and then I’m out of here. I’m so sick of this place, I want to go away. I have no clue whether it’s going to help’ (Claudia). On the other hand, the worsening of the situation variantly instigated the participants to do something about it (e.g., take drastic choices):

‘The moment I noticed I was taking it out on my daughter (…) there began to ring a bell, alarming me: that child can’t do anything about the situation you are in. Maybe it is better if you just leave [your husband]. Or you either accept that you’re in this situation and carry on. (…) So, I eventually decided to divorce.’ (Sandra)

Apart from the worsening of the situation, the *encouragement of others* (e.g., close relatives, a police officer, a psychiatrist, etc.) rarely incited participants to take action in order to ameliorate their well-being. Michelle testified how the police’s advice incited her to draw boundaries towards her abusive, alcoholic ex-husband:

‘Eventually the police helped me by saying: “Madam, I know it is a difficult situation, you want to keep your hands clean because that man has visiting rights. But it is also your duty as a mother to protect your children.” I don’t know who it was but I’m very grateful to him because it is because of that person I said a certain moment: “Indeed, I have to protect my children”.’ (Michelle)

#### In my own interest

Taking action was not only initiated by external motives but was also led by participants’ own needs. Participants typically explained how they took decisions in their own interest, regardless of their environments’ support. These decisions often concerned *important life choices* such as partner and work choice. This was especially clear in the case of Laura. She chose to study longer than was allowed by her parents. Not having children was a deliberate choice, she limited the contact with her father, she decided to keep working, even though she felt bad, etc.: ‘[My husband’s] advice was to stay home and to get me put on sick leave by the doctor. Well, that’s the solution to have me sitting next to you [her husband] on the couch. I don’t know, I don’t think so. According to me, it is better to stay busy. It gets my mind off problems; it gives structure to my week and day.’

Furthermore, every participant described their *own solutions to handle difficulties*. In contrast with the above-described life choices, these solutions were rather short-term coping strategies (e.g., taking long walks, watching a movie, etc.) that offered temporary relief for experienced pain or trouble. ‘Whenever I feel it [the agitation] coming, I try to go for a walk and I just keep walking until I got a rhythm, no longer think [about my troubles] and have the feeling of being calmer.’ (Christina).

Participants typically described *therapy as a tool* they actively sought to manage their problems. As Laura described, therapy is ‘a useful tool *to get back up’*. Participants typically expected therapy to offer tools to handle problems and ‘turn their complaints into strengths’. This is in contrast with the participants who expect the therapist to intervene in several ways to alleviate their suffering (C1; passive position in therapy). Participants rarely sought help outside of therapy. Patrick had previously found solace in self-help books:

‘I do read many, uhm, inspiring books and… I only read non-fiction… and mainly about uhm… personal development and spirituality and books that inspire me to provide insight into my thoughts and my own life. A bit more insight and uhm, more focused on self-help.’

## Discussion

The present study investigated the appearance of agency in clients’ narratives prior to treatment. Using consensual qualitative research (Hill, 2012), we constructed three overarching categories (can’t get a grip on problems, noticing own involvement and taking action) between which the participants oscillated. All themes were subdivided in 14 sub-categories.

All participants narrated about problems they don’t have a grip on (C1). This first category shows parallels with established literature on client agency, suggesting that clients present themselves as lacking agency at the outset of therapy (Toivonen et al., 2020; Wahlström, 2006). On the one hand, participants presented themselves as lacking agency by attributing their problems to *general, external circumstances* and the *malevolence of specific others*, as such situating the locus of control outside themselves (Rotter, 1966). The latter subtheme shows interesting parallels with the helplessness Vanheule and Hauser (2008) described as preceded by a disturbing encounter with enigmatic intentions of a significant other. On the other hand, participants described situations where they experience *difficulties within themselves*. Interestingly, they described situations where ‘they don’t recognise themselves’, which indicates that clients are suffering from experiences, feelings and actions that don’t fit their image of themselves and thus are experienced as ego-dystonic. Despite the disrupted feeling of self-continuity, participants formulate their problems as *static* and *unchangeable* which seems to reflect their feelings of hopelessness and despair and as such is in contradiction to one of the basic human needs for control and orientation (Epstein, 1990). Moreover, the participants’ formulations of themselves as lacking agency could be considered as examples of what Schafer (1993) calls disclaimed actions: ‘Through such disclaiming, one simply appears as the victim or witness of happenings whose origins and explanations lie entirely outside one’s own sphere of influence’ (Schafer, 1993, p. 242). Building on Schafer’s (1993) statement that change throughout therapy occurs in telling and retelling narrative claims of agency, we hypothesize that reconstructing narratives in such a way that initially disclaimed thoughts, desires and actions are reformulated as belonging to themselves, will incite clients’ involvement in therapy and open room for change.

In line with earlier research (Cauwe, Dulsster, Vanheule & Norman, 2018), the current study found that clients already noticed an involvement in their difficulties *before* entering therapy. Moreover, whereas participants typically noticed their involvement in the problems at some point, the extent to which it was in the foreground and the depth with which it was questioned and elaborated varied between participants. This raises several questions and hypotheses concerning the role of noticing an involvement in the therapeutic process. Firstly, we wonder how the action of going in therapy might stem from and/or enhance the questioning regarding one’s involvement in the presented problems. Since participants already noticed an involvement during the clinical interviews prior to therapy, we ask ourselves whether therapeutic effects might be initiated before entering therapy, instigated by the mere telling of the participants’ life stories to the interviewer-psychologist. Secondly, building on the study of Hauser, Allen and Golden (2006) we expect clients who assume responsibility for their involvement in the experienced suffering to be more agentic and resilient. If so, promoting the acknowledgement and questioning of clients’ involvement in their suffering could be considered paramount to initiate therapeutic progress. It is, however, unclear how this takes form during therapy. Analyses of therapy sessions are needed to further investigate this phenomenon.

Participants narrated during their interviews how they have already taken certain actions, either alone or motivated by previous (therapeutic or other) encounters. Nonetheless, all participants sought further professional help as their own means could not elevate their suffering. As Cauwe et al. (2018, p. 132) state: ‘Clearly, some patients have already worked very hard to solve their problems before actually starting therapy’. These results point to the clinical importance to be aware of actions clients have already taken before entering therapy. We may ask ourselves how the actions during the therapeutic process differ from actions taken prior to therapy. Investigating how clients take action during the therapeutic process might deepen our understanding about the role of agency during the therapeutic treatment. Based on our analyses, a relation between noticing an own involvement and taking action could not be concluded. Rather, taking action seemed prompted by the worsening of circumstances and encouragement of others. These results may be explained by the fact that analyses were carried out on interviews prior to treatment.

We observed a dynamic interaction within clients regarding their narrative displays of agency: participants oscillated between the experience of loss of grip on problems (C1) and respectively noticing their own involvement (C2) and taking action (C3). The fact that we observed dynamic interaction within participants adds depth to the conception of agency that was previously often conceived in a quantitative manner (e.g., Adler, Skalina & McAdams, 2008; Adler, 2012; Hobbs & Mclaren, 2009; Irving, et al., 2004). The fluctuation in client agency before entering treatment propounds a multi-layered conceptualisation of agency. In line with Safran (2016) we found a dialectic between accounts of agency (i.e., taking action and questioning one’s involvement) and nonagency (i.e., experiencing problems as uncontrollable). The latter point aligns with Schafer’s conceptualisation of the unitary agent, where ‘unitary’ does not propound the idea of an integrated, harmonious, self-conscious agent. Quite the contrary, the unitary agent refers to

‘one person doing lots of things at once, that is, performing very complex actions while defensively and in self-contradiction restricting his or her being consciously aware of what is being done and why’ (Schafer, 1993, p. 64).

This ambivalence and self-contradiction is clearly embedded in a psychoanalytic take on psychic functioning that propounds a divided and thus an ambivalent, multi-layered and dynamic subject (Dulsster et al., 2019; Schafer, 1993; Vanheule and Verhaeghe, 2009). Hence, building on the current study, we may conclude that agency is multi-layered, ambiguous and constantly shifting, yet belonging to one person, a unitary agent with a conflicted, divided psyche.

### Strengths, Limitations and Future Directions

The present study is one of the first in constructing a data-driven, qualitative account on client agency prior to therapy. In doing so, it deepens our understanding on how client agency comes about and fluctuates at the onset of therapy. By focusing on a bottom-up comprehension of how agency prior to therapy appears in clients’ narratives, this study was not able to create a fine-grained, idiosyncratic account on agency for each singular participant. Future research on case-level would offer an interesting deepening of our findings (Stiles, 2007).

Since a lack of agency is predominantly present in a population of depressed clients, we opted to randomly select participants in an RCT-study on clients with MDD (Hirsch, Sirois & Lyness, 2011). The included participants showed comorbidities with anxiety disorders and personality disorders, which makes the selected cases representative for clients in primary care settings (Hirschfeld, 2001). However, the current sample does not allow us to draw conclusions on how agency might appear in other psychopathologies like psychosis or substance abuse. Moreover, since MDD is more prevalent in women (Schuch et al., 2014), the majority of our participants are female. Future research concerning agency would benefit from sampling participants with different gender, psychopathologies and ethnic backgrounds.

Additionally, we did not select purposefully within the RCT sample. As a result, a considerable proportion of the selected participants had previous experience with psychotherapeutic help, including therapeutic experiences in the distant past, consulting multiple psychologists and psychiatrists, as well as only attending one therapy session. However, as the search for therapeutic help is primarily prompted by an experienced lack of agency (Wahlström, 2006) and given all included participants voluntarily sought assistance, we considered participants with previous therapeutic experiences as equally appropriate to investigate agency before the onset of therapy. It may nevertheless be that as a result of their earlier experiences some participants had a different attitude towards the possibility of therapy. Future research on how agency appears prior to therapy, would benefit of purposeful sampling that excludes participants with earlier therapeutic experiences.

Lastly, it is important to note that all participants included in the study took the step to therapy. As such, the results are not directly applicable to a population that did not seek therapeutic help. Research in persons who do not request therapeutic help might be interesting, yet difficult to operationalise.

## Conclusion

In their narratives, participants oscillated between having no grip on problems, noticing an involvement and taking action. This supports a multi-layered and dynamic take on agency in which there is a continuous dialectic between agency and nonagency. More fine-grained research on this dialectic is needed.
